# Morphometric Evaluations and Yields from Commercial Cuts of Black Pacu *Colossoma macropomum* (Cuvier, 1818) in Different Body Weights

**DOI:** 10.1155/2021/3305286

**Published:** 2021-12-29

**Authors:** Jucilene Cavali, Beatriz Andrade Nóbrega, Jerônimo Vieira Dantas Filho, Elvino Ferreira, Marlos Oliveira Porto, Rute Bianchini Pontuschka, Rilke Tadeu Fonseca de Freitas

**Affiliations:** ^1^Universidade Federal de Rondônia, Programa de Pós-Graduação em Ciências Ambientais, Av. Norte Sul-Nova Morada, CEP 76940-000, Rolim de Moura, Rondônia, Brazil; ^2^Universidade Federal de Rondônia, Departamento de Engenharia de Pesca, Rua da Paz, 4376-Lino Alves Teixeira, CEP 76916-000, Presidente Médici, Rondônia, Brazil; ^3^Universidade Federal de Lavras, Programa de Pós-Graduação em Zootecnia, CEP 37200-000, Lavras, Minas Gerais, Brazil

## Abstract

For the Amazon, it is important to encourage the production of native fish, since there are several species of zootechnical interest. For this, it is necessary to know the species since its acquisition, production, processing, and marketing. Therefore, the aim of this study was to analyze the yield, correlations, and profitability of different cuts of black pacu (*Colossoma macropomum*) in five weight categories. Data from 220 black pacus were obtained, with a weight range of 2725 ± 1975 g. Yields of commercial cuts and morphometric measurements were evaluated in five weight classes. Kruskal–Wallis test (*α* = 0.05) was used to compare the average income. And, to verify the correlation between the morphometric measures and the yields, Spearman's correlation was performed (*α* = 0.05). To obtain the profit of the weight classes due to the cut produced, an economic analysis was carried out considering the cost of buying the fish. Yields of fillet, ribs, and steak were higher in classes 3 and 4, while in the same classes, the yield of residues was the lowest. Despite classes 1, 2, and 5 showing lower yields, the economic analysis presented that the production of fillet and ribs was profitable. The measures of total length and standard length have a high positive correlation with the yield of meat in all classes, as well as the average circumference and caudal circumference in classes 1, 2, and 4, which can be used to determine the yields of this commercial cut.

## 1. Introduction

Aquaculture worldwide has stood out as an activity producing quality food, mainly in fish farming, with the creation of fish of commercial importance [1, 2]. In Brazil, it is not different [3], the country had a fish production of 722,560 tons in 2018, and of these, 287,910 tons were of native fish. Several species of native fish have been considered with productive potential, due to rapid growth and market acceptance, with black pacu standing out among the species [4]. Black pacu *Colossoma macropomum* (Cuvier, 1818) is the most produced native species in the country, and the state of Rondônia is the leader in the production of this species, reaching 65,500 tons produced in 2020 [5]. Native to the Amazon and Orinoco river basins, the black pacu has characteristics favorable to cultivation due to its rusticity and rapid adaptation to captivity [6, 7]. The importance of the growth of fish farming in the Amazon and the conquest of new markets, particularly the external one, certainly depends on the link with the fish processing process, in view of the offer of products that better meet the needs and convenience of consumers [4].

Studies on the body weights and yields of fish are of great importance from an economic point of view, because through these, it is possible to estimate productivity, both for the fish farmer and for the industry [8, 9]. In order to subsidize fish farming, it is necessary to generate recent and reliable data about the yield of the main species exploited in the state and, thus, ensure the planning and growth of the industrial fish sector [10]. Due to the majority of black pacu production still being marketed as whole gutted fish and the lack of standard cuts, the implementation of a fish classification system would assist in the production process, adding value to the final product. The classification system consists of grouping similar characteristics into classes, for example, the categories of sex, age, and weight, forming homogeneous categories related to productivity [11]. Another important tool for the productive characterization of cuts is through the correlation coefficients. Correlation is the standardized measure of the relationship between two variables and indicates the strength and direction of the linear relationship between these variables [2]. Correlating yields with morphometric measurements can be an important procedure to determine slaughter weights and body yields, without the need to sacrifice the animal [12].

There are few studies evaluating the yield of black pacu processing and the morphometric correlations associated with these yields, as well as no studies were found on the economic viability of commercial black pacu cuts in different weight classes. Thus, the aim of this study was to analyze the yield, correlations, and profitability of different cuts of black pacu (*C. macropomum*) in five categories of body weight.

## 2. Material and Methods

The study was approved by the Ethics Committee on Animal Use, Universidade Federal de Rondônia (UNIR). Sample collections were carried out in two fish processing units in Rondônia state, Brazil. We emphasize that the fish processing units visited are certified by the Municipal Inspection Service and the Federal Inspection Service.

Sample collections were carried out between September 2017 and March 2019 and, in all, data were obtained from 220 specimens of black pacu (*Colossoma macropomum*), with weight ranging 2725 ± 1975 g. To avoid data outliers, the sampled fish were selected from fish farms previously characterized by the fish processing unit. Fish samples were excluded from lots of production systems that adopted production management different from that commonly adopted in fish farms that adopted sanitary control. Examples of outliers are reports of parasitic infestations, deaths due to high stocking density, malnutrition, cultivation in canvas or net tanks, among others.

For the morphometric evaluations and yields of commercial cuts, they were analyzed in different weight classes. In the first moment of the study, the weight classes were established in relation to the body weight of the animals linked to the yields of commercial cuts, valuing the formation of homogeneous categories. The weight range in each class was determined based on the empirical knowledge of the fish processing industry, which observed fluctuations in the yields of the cuts depending on the slaughter weight, with the following body weight classes for the black pacu being preestablished:

- Weight class 1: <1.2 kg
- Weight class 2: from 1.21 to 1.8 kg
- Weight class 3: from 1.81 a 2.4 kg
- Weight class 4 from 2.41 a 3.5 kg
- Weight class 5: >3.5 kg

In the production unit, before the fish entered the production line, weighing, body measurements, and fish identification were carried out using labels attached to the operculum, which accompanied the waste boxes and cuts until the end of the production line, allowing traceability of the fish samples. Then, the following morphometric measures were evaluated: total length (CT), from the anterior end of the head to the end of the fish's tail; standard length (CP), from the anterior end of the head to the end of the caudal peduncle; head length (CC), between the anterior end of the head and the caudal border of the operculum; cranial circumference (CirC), measured at the end of the operculum; medium circumference (CirM), taken in the largest part of the body; and caudal circumference (CirCau), measured in the animal's caudal region.

It is important to inform, for the formalization of the study, that the methods of insensitivity and slaughter were not the responsibility of those surveyed, but of the fish processing unit, which has approved certification (by the responsible inspection) for fish insensibility and slaughter. However, researchers were informed that the fish were stunned by thermal shock and slaughtered with a cut in the jugular, in the carotid artery.

**Figure 1:**
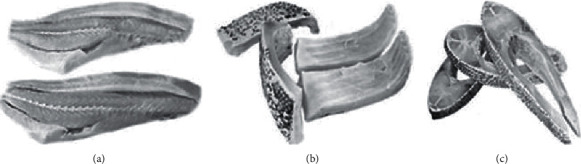
Initial processing of black pacu (*Colossoma macropomum*) and cut commercialized in the Rondônia state, Amazon, Brazil. (a) Fillet with skin, (b) ribs, and (c) steak.

The first stage of processing was performed on the evisceration table, with the procedure of removing the head through the section at the junction with the spine, flaking, and removing the viscera. While the fish were destined for the commercial cut, the saw cut was made with a bench band saw. For fillet and ribs cuts, the spine was still removed on the evisceration table, and later, the intramuscular spines were removed and the cuts were separated on the cutting table (Figure 1).

The thorns removed in the fillet have a large amount of meat, being used in the manufacture of the pulp (mechanically separated meat: CMS) of black pacu by averages of grinding. Throughout the process, all waste and products were packaged in boxes and weighed on a digital scale with an accuracy of 0.05 g. The fractions of the head, organs, internal fat, spine, and fins are given as the residue of the slaughter of the black pacu. Through equation (1), the yields of fillet, ribs, CMS, fillet, and residues were calculated.

(1) $$\begin{array}{c} R\%=\frac{p}{\text{PT}}\times 100, \end{array}$$

where R% is the yield; *p* is the weight of the sample; and PT is the total weight of the fish.

For statistical analysis, Shapiro–Wilk and Levene tests (*α* = 0.05) were conducted to verify normality and homogeneity. The Kruskal–Wallis nonparametric test (*α* = 0.05) was chosen to compare the averages followed by the standard deviation of income between weight classes. To check the correlation between the morphometric measures and the yields, Spearman's correlation coefficient test was performed (*α* = 0.05). The software used to carry out the statistical analysis was the Genes Program made available by the Universidade Federal de Viçosa (UFV), version 13.3 [13]; it is worth mentioning that the statistical program RStudio was linked to facilitate the interpretation of the results. Also, an economic analysis was carried out to obtain profit in each of the classes due to the cut produced, considering only the cost of fish acquisition, through equation.

(2) $$\begin{array}{c} \text{profit}=\frac{\left(\text{PT}\times R\%\times p\right)-\left(\text{PT}\times v\right)}{\text{PT}}, \end{array}$$

where PT is the total weight, *R*% is the cutting yield, *p* is the selling price of the cut by the fish processing unit, and *v* is the amount paid by the processing unit to the producer.

## 3. Results

The yields of commercial black pacu cuts differed between weight classes (*p* < 0.05), with classes 3 and 4 showing the highest yields for the three cuts evaluated. The yield of CMS varied between weight classes, reaching 7.35% in weight class 2, as fish weighed between 1.21 and 1.8 kg. The residues' presented yields are inversely proportional to commercial cuts, being less representative in classes 3 and 4 (Table 1). The fillet evaluated in this study is made with skin and without the intramuscular spines; these spines that discourage the consumption of black pacu but with the withdrawal have great acceptance. Black pacu between 1.81 and 3.5 kg presented fillet yield of up to 26.20%. Following the results of the fillet, the rib yield was higher for fish in classes 3, 4, and 5, from 1.8 to 3.5 kg, reaching 22% of the animals' body weight. This is attributed to the fact that black pacu, like round fish in general, has a large abdominal cavity as an adult.

Tambaqui meat has an excellent yield, especially the steak, as the viscera, the animal's head, and the scales are removed for the production of this commercial cut. Along with the edible parts, the spines, steak, and ribs are in the steak, guaranteeing a yield of 58.9 and 56.4% for animals of classes 3 and 4, respectively. The average yield of residues from the production of fillet and ribs was 48.90%, while the yield of residues from the production of steak was 45.05% (Table 1). This averages that almost half of the amount paid for the whole fish is actually being paid for the waste, and taking advantage of it is the economically and environmentally viable alternative. However, CMS represents on average 6.71% of the total black pacu weight, being a very commercialized by-product. Some government programs have encouraged the inclusion of fish in school meals, a market niche that absorbs a large part of the CMS produced by agribusinesses.

The fish in body weight classes 3 and 4 presented higher economic returns to the fish industry in the three commercial cuts evaluated, while animals in classes 1 and 2, this profit is 30% lower for fillet, 22% lower for ribs, and up to 78% lower to steak. Weight class 5 is still profitable for the production of fillet and ribs; however, for steak, the profit is the lowest among all weight classes evaluated (Table 2). Black pacu is commercialized at the processing unit for the weight of the whole fish, with an average regional value of R$ 5.00 kg^−1^, and the processing industries prioritize animals with a minimum weight of 1.4 kg.

The bone-free fillet is the most required cut by the most demanding markets and consumers since intramuscular spines discourage the consumption of round fish, such as black pacu. With a pleasant and very nutritious flavor, it has an average commercial value of R$ 20.75 kg^−1^. Nonetheless, ribs are also much appreciated and used as a snack throughout the North region, being commercialized with an average value of R$ 19.00 kg^−1^. Ribs are a cut with “Y” spines, being less required in the fish industry. Still, they are tasty and nutritious, with the cut of the lowest average commercial value, of R$ 9.90 kg^−1^.

When considering the commercialization value of the fillet, ribs, and CMS, it was observed that the black pacu of classes 3, 4, and 5 generated the highest profit of R$ 4.88, R$ 5.14, and R$ 4.78 kg^−1^, yielding up to R$ 18.00 in a 3.5 kg fish. Classes 1 and 2 are the ones that presented the lowest profit when compared to the others, of R$ 4.06 and R$ 3.82, respectively. However, it is still possible to indicate the production of fillet and ribs. On the other hand, the profit for the fish in weight classes 1 and 2, as well as in weight class 5, is very low, which makes the production of this cut unfeasible. The profit of R$ 1.99 for fish in class 3 and R$ 1.71 in weight class 4 is lower than that observed for fillet and ribs, indicating that the production of steak must be directed to meet specific demands.

The fillet yield and morphometric measurements presented a significant correlation coefficient in all classes of body weight, except in weight class 4 (Table 3). Correlations were moderately negative, with emphasis on CT and CirM measurements. The rib yield and morphometric measurements also presented a negative correlation coefficient in all body weight classes, with the exception of weight class 4 (Table 4). All significant correlations with this cut were negative, with some measures showing a high correlation, such as CP in class 2 and CirCau in class 3. The yield of the post and CT and CP measures presented a high positive correlation coefficient in all classes of body weight, while CC was not significant in any class. In wight classes 1 and 2, the measures CirC, CirM, and CirCau also had a high positive correlation with the performance of the meat, while in class 4 the measures CirM and CirCau (Table 5).

The commercial cuts' yield and the CT and CP measurements presented a high correlation coefficient in all weight classes, as well as CirM and CirCau in weight classes 1, 2, and 4. In the commercial cuts, only the scales, head, viscera, and tail are removed, ensuring greater yield. As a result of the utilization characteristic of the entire length of the fish, the great correlation of CT and CP with the yield of the fish was guaranteed. CirC, CirM, and CirCau also obtained a great correlation with the cut due to the use of the entire circumference of the fish.

## 4. Discussion

The fish processing and the development of value-added products are viable alternatives to improve profitability, by expanding your market and increasing profitability [14]. The yield allows the producer to plan the amount of fish that will be needed to deliver the slaughter, conditioning the aspect of the products and sales strategies [15]. Gutted whole fish is the most commercialized form of fish in the Amazon [12, 16] and, according to Buzollo et al. [9], this part of the fish can yield about 60% of the total weight in tropical fish. However, the fillet can present a higher percentage of yield and is considered the noblest part of the fish, being very appreciated for the flavor and practicality of the preparation. Lima et al. [17] evaluating fillet yield in three weight classes obtained a yield of 57.5% in black pacu below 500 g, 44.6% with a weight between 1 and 1.5 kg, and 48.7% for black pacu above 2 kg. However, these yields went to fillet with skin, spines, and ribs; this type of fillet is known in the industry as a “headless band.”

It is believed that the fillet yield of weight classes 1 and 2, below 1.2 kg and 1.21 to 1.8 kg body weight, was lower due to the residues being more representative in fish in these classes (*p* < 0.05) and also for the variation in the removal of intramuscular spines, which carry part of the muscle tissue of the fillet. Although the black pacu fillet has spines in large quantities and in the shape of a “Y”, this part can be cut into strips to solve the problems of intramuscular spines in the consumer market [18]. In the processing fish industry, the fillet with skin, because it contains a large amount of meat near the spines, is minimally ground and used for the production of the CMS of black pacu [19].

Dantas Filho et al. [2] and Roubach and Saint-Paul [20] highlight another cut with great importance as a culinary product, the black pacu ribs, because the fish has long ribs and soft meat. Usually, the black pacu ribs are commercialized in small strips, constituting a typical dish in restaurants in the northern region of Brazil [18]. Cartonilho and Jesus [21] describe a rib yield of 19.64% for black pacu with an average weight of 1.54 kg, while the yield mentioned by Fernandes et al. [22] reached 17.4% in black pacu with 1.45 kg of average body weight. For the black pacu, specimens with a smaller head and body shape 1 : 1 (length: height) are preferred, so that a higher proportion of ribs and less waste (head and fins) are obtained.

The average weight is important in the commercial cut definitions in the processing performed by the industry, since it influences the beheading, evisceration, and general cleaning operations, by manual or mechanized methods [15]. The weight of the fish also influences the adequacy and yield of the meat when preprocessed in the form of a clean body, steak or fillet, in addition to the speed of cooling and freezing in the fish industry [9]. Much of the excess fat in round fish, such as black pacu, is deposited in the abdominal cavity, which results in great loss of the yield of edible parts or lower yield in the final processing of the fish [22]. Therefore, there is a need to ensure an ideal slaughter weight class, so that the fish are in the appropriate growth range for the lowest fat accumulation and highest yields.

Generally, the residues are a surplus part of the activities of the fish processing fish industry, being classified as gaseous, liquid, or solid components and which, when released into the environment without proper treatment, can cause serious changes in the characteristics of air, water, and soil, becoming harmful to the aquatic and terrestrial ecosystem [23]. Methods involving processes of distribution, collection, destination, treatment, and processing, seeking efficient and inexpensive methodologies that result in environmental impacts on the smallest possible scale, have been studied [19, 24, 25]. Fernandes et al. [12] describe the viscera, scales, and bones, for example, as raw materials for the manufacture of flours, silages, and fish oils, commonly used in animal feed, while the carcass containing residual fillet meat is subjected to processes to obtain of the fish CMS, the main ingredient in the manufacture of breaded and sausages, which are highly appreciated in human food and with excellent added value.

An alternative used for the destination of nonedible parts is the production of organic fertilizer. A source of organic carbon from fish, it is rich in several amino acids and enzymes readily available to plants that improve the translocation of the absorbed nutrients, promoting better maturity uniformity [23]. Another alternative employed is the ensilage of waste. Several authors emphasize the great potential that silage presents, which can be used in fish farming due to the similarity with the raw material that gave rise to it, providing quality nutrients, especially proteins; other characteristics that must be mentioned are the high digestibility and the lower cost in relation to fish meal [26–29].

Maximizing production, combined with the quality of the final product (fish with a higher percentage of muscle tissue), is a requirement of fish processing units and consumers themselves [9, 30]. The knowledge of the portion of edible parts and the different forms of presentation of the cuts of the fish, as well as the quantity that will be part of the processing by-products, allows the logistic planning of the production and the necessary calculations for the evaluation of its viability and economic efficiency [9, 16]. Currently, the commercialization of processed fish occurs mainly in the form of whole animals and only gutted, mainly in native species such as pacu (*Piaractus mesopotamicus*) black pacu (*Colossoma macropomum*) and pirapitinga (*Piaractus brachypomus*) [18]. This form of commercialization limits consumption, mainly due to the lack of practicality and standardization of the product with regard to the characteristics of taste, presence or absence of spines, preparation, and nutritional value [19].

The productive sector of fish farming can only consolidate and become competitive with other meat-producing industrial segments once the various technological problems related to slaughter, handling, processing, storage, commercialization, distribution [31], and quality management of value-added products are solved [17]. According to Bombardelli et al. [32], these problems are mainly responsible for the reduction of meat quality, expiration date, and fish consumption, and the modernization process will allow a greater added value to products and by-products, in addition to allowing popularization in the consumer market.

Studies of the body weights and yields of fish are of great importance from an economic point of view, because through the body yields, an estimate of productivity can be made, both for the fish farmer and for the fish processing industry [11, 26]. One of the indirect ways of characterizing the carcass is through morphometric measurements, which can be a very important procedure to estimate body weights and yields, without the need to slaughter the animal [12]. Morphometric measurements directly contribute to a description of the fish's body shape, which can vary depending on the characteristics of each species, in addition to influencing body weight and meat yields [8].

The correlation is one of the ways to verify the interrelationship between relevant characters [9]. Correlations between body weights and yields with morphometric measurements have been the object of study of several studies on several species of fish such as piracanjuba (*Brycon orbignyanus*) [12], paiche (*Arapaima gigas*) [33], and tilapia (*Oreochromis niloticus*) and black pacu [29].

The correlations observed between the fillet yield and the morphometric measurements, as well as the rib yield and the measurements, were negative. These negative correlation results may be associated with the fact that black pacu has a large abdominal cavity and greater facility in storing abdominal fat [23]; larger black pacu may have better meat yields; however, it brings with it a high accumulation of visceral fat. So, with weight gain, greater measurements of CirM and CT do not necessarily indicate a higher proportion of fillet and ribs, but it may be associated with visceral fat. Buzollo et al. [9] observed that, for fillet yield, body width was the most suitable measure for determining this yield (0.410), and the greater the body width, the greater the fillet yield. For rib yield, the same author points out that morphometric measurements are not indicated to estimate the same. Likewise, Alberto et al. [8] believed that one or more unmeasured morphometric measures may influence the yields studied, or else that the values of these variables depend more on the efficiency of processing than on the intrinsic characteristics of the raw material, such as the shape of the body and its relations.

Studies to select fish with better fillet yield are concentrated on the use of body measurements, and many of these studies have presented a low linear relationship between fillet yield and body characteristics, but a high relation with fillet weight [33, 34]. A high correlation of measures is expected to increase together over time, such as the association of total weight with fillet weight. Rare cases, such as skinny fish “facão” or in the reproductive period, can interfere in the high correlation of characteristics that are naturally directly proportional [33].

## 5. Conclusions

The weight classes exerted significant differences in the black pacu (*C. macropomum*) evaluated characteristics. The yields of fillet, ribs, and steak were higher for fish between 1.81 and 3.5 kg, while the yield of residues was inversely proportional. The economic analysis proved to be profitable for the production of fillet and ribs in all weight classes and for the cut put only in weight classes 3 and 4, with fish between 1.81 and 3.5 kg. The production of CMS, biofertilizer, and silage are alternatives adopted by the processing units for the disposal of residues. The CT and CP measures have a high positive correlation with the fish stock yield in all classes, as well as the CirM and CirCau in weight classes 1, 2, and 4, which can be used to determine the yield of the fish stock. The knowledge of the weights that provide less residues production helps producers to remove animals from the production system at the appropriate time, with lower maintenance costs and reducing impacts on water quality. These animals generate better income for the fish industry, guaranteeing quality and standardization to the final consumer.

## Figures and Tables

**Table 1:** Yields of commercial cuts, CMS, and residues of black pacu (*Colossoma macropomum*) in relation to each weight class.

Weight classes	Yields (%)						
	Fillet with skin	Ribs	CMS	Resídues^1^	Steak	Resídues^2^	*n*
1 (<1.2 kg)	22.04 ± 3.43^b^	19.84 ± 2.54^b^	7.00 ± 1.19^ab^	51.11 ± 5.20^a^	54.15 ± 5.38^bc^	45.84 ± 5.66^ab^	45
2 (1.21 to 1.8 kg)	21.33 ± 3.32^b^	19.13 ± 2.45^b^	7.35 ± 1.25^a^	52.18 ± 5.31^a^	54.34 ± 5.40^bc^	45.66 ± 5.64^ab^	45
3 (1.81 to 2.4 kg)	26.20 ± 4.07^ab^	19.90 ± 2.55^ab^	6.44 ± 1.09^ab^	47.44 ± 4.82^b^	58.89 ± 5.85^a^	41.10 ± 5.08^b^	45
4 (2.41 to 3.5 kg)	25.68 ± 3.99^ab^	22.00 ± 2.82^a^	6.21 ± 1.05^b^	46.09 ± 4.69^b^	56.38 ± 5.60^a^	43.62 ± 5.39^b^	50
5 (>3.51 kg)	23.76 ± 3.69^b^	22.01 ± 2.82^a^	6.55 ± 1.11^ab^	47.67 ± 4.85^b^	50.96 ± 5.06^c^	49.04 ± 6.06^a^	30

If there are averages followed by different letters (a, b) in the columns, they are different from each other by the Kruskal–Wallis test (*p* < 0.05). ^1^Residues from the production of fillet and ribs. ^2^Residues from the production of meat.

**Table 2:** Economic return of commercial black pacu (*Colossoma macropomum*) cuts in different weight classes.

Weigh classes	Profit^*∗*^kg^−1^ of black pacu (R$)			
	Fillet with skin	Ribs	CMS	Steak
1 (<1.2 kg)	1.83	1.65	0.58	0.43
2 (1.21 to 1.8 kg)	1.70	1.53	0.59	0.68
3 (1.81 to 2.4 kg)	2.43	1.85	0.60	1.99
4 (2.41 to 3.5 kg)	2.45	2.10	0.59	1.71
5 (>3.51)	2.17	2.01	0.60	0.16

^*∗*^Considering the purchase price of black pacu at R$ 5.00 kg^−1^.

**Table 3:** Correlations between fillet yield and morphometric measurements in relation to the different weight classes of black pacu (*Colossoma macropomum*).

Weight classes	Fillet yield					
	CT	CP	CC	CirC	CirM	CirCau
1 (<1.2 kg)	−0.641^*∗∗*^	−0.606^*∗∗*^	−0.758^*∗∗*^	ns	−0.683^*∗∗*^	−0.517^*∗∗*^
2 (1.21 to 1.8 kg)	−0.504^*∗*^	ns	ns	−0.643^*∗∗*^	−0.612^*∗∗*^	−0.508^*∗∗*^
3 (1.81 to 2.4 kg)	−0.637^*∗∗*^	−0.617^*∗∗*^	−0.422^*∗*^	ns	−0.411^*∗*^	ns
4 (2.41 to 3.5 kg)	ns	ns	ns	ns	ns	ns
5 (>3.51 kg)	−0.418^*∗*^	−0.489^*∗*^	ns	−0.451^*∗*^	−0.449^*∗*^	ns

^*∗*^Significant (*p* < 0.05); ^*∗∗*^significant (*p* < 0.01); ns = not significant; CT: total length; CP: standard length; CT: head length; CirC: cranial circumference; CirM: medium circumference; CirCau: caudal circumference.

**Table 4:** Correlations between the rib yield and morphometric measurements in relation to the different weight classes of black pacu (*Colossoma macropomum*).

Weight classes	Rib yield			
	CT	CP	CC	CirC	CirM	CirCau
1 (<1.2 kg)	−0.740^*∗∗*^	ns	ns	ns	−0.757^*∗∗*^	−0.617^*∗∗*^
2 (1.21 to 1.8 kg)	ns	−0.892^*∗∗*^	−0.592^*∗∗*^	−0.641^*∗∗*^	−0.497^*∗*^	ns
3 (1.81 to 2.4 kg)	−0.579^*∗∗*^	−0.472^*∗*^	ns	ns	−0.758^*∗∗*^	−0.802^*∗∗*^
4 (2.41 to 3.5 kg)	ns	ns	ns	ns	ns	ns
5 (>3.51 kg)	−0.465^*∗*^	−0.513^*∗∗*^	ns	−0.571^*∗∗*^	−0.534^*∗∗*^	ns

^*∗*^Significant (*p* < 0.05); ^*∗∗*^significant (*p* < 0.01); ns = not significant; CT: total length; CP: standard length; CT: head length; CirC: cranial circumference; CirM: medium circumference; CirCau: caudal circumference.

**Table 5:** Correlations between the steak yield and morphometric measurements in relation to the different weight classes of black pacu (*Colossoma macropomum*).

Weight classes	Steak yield					
	CT	CP	CC	CirC	CirM	CirCau
1 (<1.2 kg)	0.885^*∗∗*^	0.926^*∗∗*^	ns	0.947^*∗∗*^	0.951^*∗∗*^	0.902^*∗∗*^
2 (1.21 to 1.8 kg)	0.811^*∗∗*^	0.705^*∗∗*^	ns	0.857^*∗∗*^	0.843^*∗∗*^	0.750^*∗∗*^
3 (1.81 to 2.4 kg)	0.749^*∗*^	0.836^*∗∗*^	ns	ns	ns	ns
4 (2.41 to 3.5 kg)	0.896^*∗∗*^	0.824^*∗∗*^	ns	ns	0.799^*∗∗*^	0.549^*∗∗*^
5 (>3.51 kg)	0.749^*∗*^	0.836^*∗∗*^	ns	ns	ns	ns

^*∗*^Significant (*p* < 0.05); ^*∗∗*^significant (*p* < 0.01); ns = not significant; CT: total length; CP: standard length; CT: head length; CirC: cranial circumference; CirM: medium circumference; CirCau: caudal circumference.

## Data Availability

The data used to support the findings of this study are available from the corresponding author upon request.
